# Prevention of transmission of *Babesia canis* by *Dermacentor reticulatus* ticks to dogs after topical administration of fluralaner spot-on solution

**DOI:** 10.1186/s13071-016-1481-x

**Published:** 2016-05-31

**Authors:** Janina Taenzler, Julian Liebenberg, Rainer K. A. Roepke, Anja R. Heckeroth

**Affiliations:** MSD Animal Health Innovation GmbH, ZurPropstei, 55270 Schwabenheim, Germany; ClinVet International, Uitsigweg, Bainsvlei, 9338 Bloemfontein, Free State South Africa

**Keywords:** Bravecto™ Spot-on Solution, *Babesia canis*, Babesiosis, Fluralaner, *Dermacentor reticulatus*, Dog, Efficacy, Prevention of transmission, Spot-on

## Abstract

**Background:**

The preventive effect of fluralaner spot-on solution against transmission of *Babesia canis* by *Dermacentor reticulatus* ticks was evaluated.

**Findings:**

Sixteen dogs, tested negative for *B. canis* by polymerase chain reaction (PCR) and immunofluorescence assay test (IFAT), were allocated to two study groups. On day 0, dogs in one group (*n* = 8) were treated once topically with fluralaner spot-on solution (Bravecto™ Spot-on Solution) according to label recommendations and dogs in the control group (*n* = 8) remained untreated. On days 2, 28, 56, 70 and 84, all dogs were infested with 50 (±4) *D. reticulatus* ticks harbouring *B. canis*, with tick in situ thumb counts 48 ± 4 h after each infestation. On day 90, ticks were removed from all dogs and counted. Prior to each infestation, the presence of *B. canis* in the respective tick batch was confirmed by PCR, and 12–16 % of ticks were found to be infected with *B. canis.* Efficacy against ticks was 99.5 and 99.3 % on days 4 and 58 after treatment, respectively and 100 % on all other days. Replacement dogs were included for any *B. canis* infected control dog (in total 19). All control dogs (*n* = 27) became infected with *B. canis*, as confirmed by PCR, performed every 7 days, and by IFAT, performed every 14 days after treatment. None of the eight treated dogs became infected with *B. canis*, as they were tested negative by PCR and IFAT throughout the study until day 112. By comparing infected dogs in the treated group with infected dogs in the untreated control group, a 100 % preventive effect against *B. canis* transmission was demonstrated.

**Conclusions:**

A single topical administration of fluralaner spot-on solution effectively prevented the transmission of *B. canis* by infected *D. reticulatus* ticks over a 12-week period.

## Findings

### Hypothesis

Fluralaner rapidly kills ticks within 12 h after tick attachment [1], and the prevention of *Babesia canis* transmission by oral treatment with fluralaner chewable tablets was already shown [2]. Recently, a new formulation of fluralaner as Bravecto™ Spot-on Solution became available [3]. Thus, the potential of topically administered fluralaner to prevent *B. canis* transmission was tested.

### Methods and study set-up

The same methodology as already described by Taenzler et al*.* [2] was applied to investigate the prevention of *B. canis* transmission by topical fluralaner treatment and, therefore, methods and study set-up are only briefly summarized below. Ethical approval was obtained by the ClinVet Animal Ethics Committee (CAEC) before study start.

### Animal details

Sixteen healthy mixed breed dogs (eight males, eight females; 1–8 years, 13.8–26.9 kg) tested negative for *B. canis* by polymerase chain reaction (PCR) and immunofluorescence assay test (IFAT), were randomly allocated to two study groups of eight dogs each. Dogs were individually housed indoors, and fed a standard commercially available dry dog food once daily; drinking water was provided *ad libitum*.

### Treatment

On day 0 (i.e. day of treatment), dogs in the treatment group were treated once topically with fluralaner spot-on solution according to the manufacturer’s label instructions. There was no evidence of mis-dosing such as spillage or run-off/drip-off in any treated animal. Dogs in the control group remained untreated.

### Tick infestations and assessments

On days 2, 28, 56, 70 and 84, all dogs were infested with 50 (± 4) adult, unfed *D. reticulatus* ticks (European origin, sex ratio 1:1). Prior to each infestation, the presence of *B. canis* was confirmed by PCR using 50 ticks of the respective batch. Tick in situ thumb counts were performed 48 ± 4 h after each infestation. On day 90, all remaining ticks on each dog were removed and counted.

### Animal health

After treatment, the health status of each animal was monitored by physical examinations on a 7-day interval and the rectal body temperature of each dog was measured thrice weekly. General health observations, noting the dog as normal or abnormal, were performed once daily starting 7 days prior to treatment until day 112 after treatment. If a dog was noted as abnormal or the rectal body temperature was above or equal to 39.4 °C, an additional physical examination was performed.

### Blood analysis

If, one or more parameters during physical examination were abnormal, a blood smear was made. Blood samples for serum analysis for antibodies to *B. canis* (IFAT) and for *B. canis* DNA detection (PCR) were collected on a 14-day interval and on a 7-day interval after treatment, respectively [2].

### Rescue treatment and replacement

Dogs confirmed positive for *B. canis* by blood smear were rescue treated using imidocarb and diminazene [2] and remained part of all health observations, but were not subjected to subsequent tick infestations. PCR and IFAT were performed on blood samples and after confirmation of a babesial infection by both analyses; these dogs were finally excluded from the study. *B. canis* positive control dogs were replaced by a *B. canis* negative replacement dog to ensure a sufficient number of control dogs for tick infestations/counting. In total, 19 replacement dogs (ten males, nine females) were used; thus, in the control group a total of 27 dogs were included.

### Efficacy evaluation

The statistical analysis was performed using the software package SAS® (SAS Institute Inc., Cary, NC, USA, release 9.3). The experimental unit was the individual dog.

The percentage of efficacy against ticks was calculated for the treatment group at each assessment time point using geometric means with Abbott’s formula:

Efficacy (%) = 100 × (M_C_ - M_T_)/M_C,_ where M_C_ is the mean number of total live attached ticks on untreated control dogs and M_T_ the mean number of total live attached ticks on treated dogs. Log-transformed counts (xi = ln(xi + 1)) of live attached ticks were used to confirm the efficacy calculation. Significant differences were assessed between the log-transformed counts of live attached ticks in the treated group at each assessment time point compared to the log-transformed counts of the untreated control group using a linear mixed model including study group as a fixed effect and block as a random effect. The two-sided level of significance was declared when *p* ≤ 0.05.

The percentage preventive effect against *B. canis* transmission for the treatment group was calculated as follows: Preventive effect (%) = 100 × (T_C_ - T_T_)/T_C_, where T_C_ is the total number of infected dogs in the untreated group and T_T_ is the total number of infected dogs in the treated group. A dog was regarded infected with *B. canis*, if it was tested positive by both IFAT and PCR. Study groups were compared using the Fisher’s exact test.

## Results

No treatment-related adverse events were observed in any of the eight dogs treated once topically with fluralaner. An efficacy against ticks at each assessment time point between 99.3 and 100 % was achieved (Table 1). At each infestation time point, 12–16 % of ticks were found to be infected with *B. canis* by PCR analysis. The infection model was regarded as valid as all 27 untreated control dogs were infected with *B. canis*, as confirmed positive for *B. canis* by blood smear, for babesial DNA by PCR analysis, and for antibodies to *B. canis* by IFAT after first or subsequent tick infestation (Table 2). Furthermore, dogs in the control group developed clinical signs referring to babesiosis as pale mucous membranes, rectal body temperature above or equal to 39.4 °C, depressed/listless general behaviour, enlarged lymph nodes and enlarged spleen. In total, 19 replacement dogs (ten male, nine female) were included in the control group throughout the study, ensuring that at each tick infestation time point, the control group consisted of eight animals, which was possible for all infestation time points except the last one on day 84. For tick challenge on day 84 only six control animals were available, from which two were tested positive by blood smear and PCR analysis on day 85 and rescue treated, so that for tick in situ thumb counting on day 86 the control group consisted of only four animals. Efficacy calculation for day 86 and day 90 were therefore calculated with four control dogs.

**Table 1:** Mean tick counts and efficacy against ticks after single topical treatment with fluralaner spot-on solution

Day 4	Mean tick counts ^a^ (control/treated) (*n*)	18.6/0.1
	Efficacy (%)	99.5 ^b^
Day 30	Mean tick counts ^a^ (control/treated) (*n*)	18.8/0.0
	Efficacy (%)	100 ^b^
Day 58	Mean tick counts ^a^ (control/treated) (*n*)	12.7/0.1
	Efficacy (%)	99.3 ^b^
Day 72	Mean tick counts ^a^ (control/treated) (*n*)	22.2/0.0
	Efficacy (%)	100 ^b^
Day 86 ^c^	Mean tick counts ^a^ (control/treated) (*n*)	23.0/0.0
	Efficacy (%)	100 ^b^
Day 90 ^c^	Mean tick counts ^a^ (control/treated) (*n*)	22.3/0.0
	Efficacy (%)	100 ^b^

^a^Geometric mean live attached ticks

^b^Log-transformed counts of ticks from the treated group were significantly different (*P* < 0.0001) from log-transformed counts of the untreated control group

^c^Control group: six dogs available for infestation, two dogs were tested positive for *B. canis* on day 85 and excluded, efficacy calculation based on four dogs

**Table 2:** Number of dogs with increased rectal body temperature (RBT) and number of dogs tested positive for *B. canis* via blood smear, PCR and IFAT

Study group	RBT	Blood smear	PCR	IFAT
	≥ 39.4 °C	[POS]	[POS]	[POS]
Untreated control (*n*)	19/27	27/27	27/27	27/27
Treatment group ^a^ (*n*)	1^b^/8	0/8	0/8	0/8

^a^Topical treatment with fluralaner spot-on solution

^b^RBT above or equal 39.4 °C was measured but not confirmed to be related to an infection with  *B. canis* as PCR and IFAT were negative throughout the study

None of the dogs treated with fluralaner spot-on solution developed any clinical signs referring to babesiosis. An increased rectal body temperature was measured in 1 of 8 treated dogs, but was not confirmed to be related to an infection with *B. canis,* as both, PCR and IFAT, were negative throughout the study. None of the treated dogs became infected with *B. canis* during the complete study duration, as confirmed by the absence of antibodies to *B. canis* in the IFAT and a negative test result for babesial DNA by PCR analysis on any of the scheduled blood analysis time points up to 4 weeks after the last tick infestation (Table 2). A 100 % preventive effect against the transmission of *B. canis* by infected *D. reticulatus* ticks was achieved after single topical fluralaner treatment (Table 3).

**Table 3:** Preventive effect against the transmission of *B. canis* by *D. reticulatus* ticks

	Untreated control	Treatment group ^a^
Infected (*n*)	27/27	0/8
Non-infected (*n*)	0/27	8/8
Infected (%)	100	0
Prevention (%)	-	100
*P*-value	-	0.0002

^a^topical treatment with fluralaner spot-on solution
